# Dietary Patterns and Cardiovascular Disease: Insights and Challenges for Considering Food Groups and Nutrient Sources

**DOI:** 10.1007/s11883-019-0770-1

**Published:** 2019-02-11

**Authors:** Linda C. Tapsell, Elizabeth P. Neale, Yasmine Probst

**Affiliations:** 1 School of Medicine, Faculty of Science, Medicine and Health, University of Wollongong, Wollongong, New South Wales 2522 Australia; 2 Illawarra Health and Medical Research Institute, University of Wollongong, Wollongong, New South Wales 2522 Australia; 3 Smart Foods Centre, University of Wollongong, Wollongong, New South Wales 2522 Australia

**Keywords:** Dietary patterns, Cardiovascular disease, Food composition, Food groups, Nutrients

## Abstract

**Purpose of Review:**

The relationship between dietary patterns and cardiovascular disease has been the subject of much research, but an important methodological consideration is the interdependence between the nutrient composition of foods and the recognition of healthy dietary patterns. This review considers some of the challenges in researching dietary patterns with implications for translation to public health promotions.

**Recent Findings:**

A number of statistical methods have emerged for analysing dietary patterns using population dietary data. There are limitations in the assumptions underpinning food categorisation, but this research is able to consistently identify foods and dietary patterns that are positively related to health. Aligned to this activity is the ongoing development of food composition databases which has its own limitations such as keeping up to date with changing foods and newly identified components, sampling of foods, and developments in chemical analytical methods. Finally, dietary patterns form the basis for current dietary guidelines and related public health–oriented programs, but the issues raised for research (e.g. food categorisation and cuisine influences on dietary patterns) can also translate to these settings.

**Summary:**

The study of dietary patterns in cardiovascular disease prevention presents with a number of methodological challenges relating to the way food groups are formed and the limitations of food composition databases. Added to this are new considerations for the environmental impact of recommended dietary patterns. Future research across the entire knowledge chain should target more accurate methods in a number of analytical areas.

## Introduction

The relationship between dietary patterns and cardiovascular health has been of scientific interest for some time [1••]. In the last century, Ancel Keys’ work on cardiovascular disease linked cholesterol levels with dietary saturated fat, although these early observations were largely undertaken in the context of a Mediterranean diet [2]. This complex situation has challenged scientists for decades, but the concentration of research on cardiovascular disease has been helpful. Despite conceptual issues that have emerged, we have gained a good understanding of the interrelationship between nutrients, foods, and whole diets and their associations and effects on disease outcomes and disease biomarkers. Underpinning this scenario is a framework for understanding nutrition, and in this case, how food consumption may ameliorate or accelerate cardiovascular disease progression.

In very basic terms, nutrition concerns the delivery of biological substances (nutrients) that influence the structure and function of the human body. Dietary patterns reflect regular choices of particular foods which in turn deliver these nutrients in a synergistic fashion [2]. It follows that the profile of food and nutrient delivery will have an impact on human biology, and these can be both beneficial and deleterious. As diseases reflect complex processes, dietary effects are likely to be confounded by a number of other factors, and clinical signs may only appear after sustained exposure. Prolonged imbalances in dietary intake, for example of excess energy, saturated fat, sodium, and added sugar, are associated with higher prevalence of chronic disease [3••]. High blood cholesterol levels remain a significant cardiovascular disease risk factor [4], but the case against saturated fat, which is associated with this biomarker has become nuanced [5•, 6, 7••]. The body of research that provides the evidence for the effects of saturated fat is varied in type (e.g. observational cohort studies versus randomised controlled trials) and design elements (such as test foods or replacement nutrients). There are arguments that the saturated fat from dairy foods may be less atherogenic, while others may stress the importance of replacing saturated fats with polyunsaturated fats rather than carbohydrates [8]. The impact of saturated fat does not occur in isolation of foods and the total diet, making studies of foods and dietary patterns an important evolutionary aspect in research on diet and cardiovascular disease.

Researching dietary patterns is not without its problems. Dietary assessment can be fraught with misreporting, and food categorisation requires critical decision making. This review considers some of the challenges in researching dietary patterns to better understand the diet-disease relationship.

### Available Methods for Defining Dietary Patterns

A number of methods can be found in the literature for assessing dietary patterns [9]. The a priori approach refers to the process of defining dietary patterns based on pre-specified criteria, such as a dietary index or score. The approach outlines the types, quantities, and consumption frequencies of foods within a desirable dietary pattern. Numerous indices exist, such as the Healthy Eating Index [10•], the Mediterranean Diet score [11], the Dietary Approaches to Hypertension (DASH) score [12], and the Dietary Inflammatory Index [13]. Diet indices may also be developed for a specific context, such as the Diet Quality Tracker [14]. The Diet Quality Tracker was developed by our study team to explore the changes in diet quality during the course of a clinical trial. It is an a priori tool, which assesses diet quality based on consumption of 10 food groups (eight core, and two discretionary food groups).

As part of the Dietary Patterns Methods Project, Liese et al. [15] applied four commonly used dietary indices, the Healthy Eating Index 2010 (HEI-2010), the Alternate Healthy Eating Index 2010 (AHEI-2010), the alternate Mediterranean Diet (aMED) score, and the DASH score, to three large population cohorts. They found a high level of consistency between the indices in classifying the quality of an individual’s diet. Importantly, a higher diet quality determined by all scores was associated with a significant reduction in mortality. These results indicated that despite some variation in the types and amounts of foods included in each index, they could consistently identify individuals with a higher quality diet, which in turn was reflected in reduced risk of chronic disease.

While a priori approaches can consistently classify individuals within a population, it is based on a pre-defined ‘ideal diet’. Thus, there are inherent limitations in applying specific tools to populations with a different dietary pattern for which they were designed (for example applying a Mediterranean dietary score to a region with a different cuisine pattern) [16]. While application of a priori scores may still have the potential to rank individuals from lower to higher quality diets, the application of a score based on a different dietary pattern (and different foods) may mean that individuals may not meet pre-defined nutrient or food cut-offs within the tool, yet their diet may be quite good quality [16]. In contrast, the a posteriori approach defines dietary patterns based on the consumption patterns of the population. Statistical methods are used to derive dietary patterns using the a posteriori approach, including ‘principal components analysis’ and ‘factor analysis’, which identify foods reported in dietary assessments that are correlated with each other [17]. Another method, known as ‘cluster analysis’, groups individuals with similar dietary intakes together into ‘clusters’ [17]. Applications of the a posteriori approach identify common dietary patterns such as ‘traditional’ or ‘prudent’ diets, which tend to be associated with reduced risk of disease, and ‘Western’ diets, which are associated with increased risk [18].

Thus, the a posteriori approach may appear more objective as it utilises the population data itself whereas the a priori approach uses pre-defined dietary criteria. Nevertheless, statistical methods such as principal components analysis still incorporate a degree of subjective decision making which may introduce bias or result in inconsistency between studies [18, 19]. In addition, while the a posteriori approach does often allow for the identification of ‘healthy’ and ‘unhealthy’ dietary patterns such as the ‘prudent’ and ‘Western’ diets, in some cases, dietary patterns identified may not always meaningfully reflect a higher or lower quality diet [16]. As such, these patterns may not always provide meaningful insights into diets that are associated with increased or reduced risk of cardiovascular disease.

In addition, differences in the dietary assessment tools used may result in inconsistency in the dietary patterns identified [20]. Within nutrition research, dietary data may be collected via a number of assessment methods, each with their own strengths and limitations for defining dietary patterns. For example, dietary data in large cohort studies is typically collected via food frequency questionnaires [21]. While these food frequency questionnaires are inexpensive and therefore suitable for use in large samples, they rely heavily on memory, which means they may be susceptible to recall bias [21]. They are also close-ended, limiting the depth of dietary information that may be collected for dietary pattern analyses. In contrast, open-ended tools such as diet histories, food records, and 24-h recalls may also be used. However, the data obtained from open-ended tools does not appear to lend itself well to the pre-defined criteria often required by a priori methods, in comparison to close-ended food frequency questionnaires. As a result, consideration of the methods used to assess dietary intake is required when designing studies to explore dietary patterns.

### Consistency of Key Foods Identified in Healthy Dietary Patterns

Despite the range of approaches applied for defining dietary patterns, they appear to be able to identify a consistent list of foods which characterise dietary patterns favourable to health, including prevention of cardiovascular disease. In their application of four existing dietary indices to three population cohorts in the Dietary Patterns Methods Project, Liese at al. [15] showed that whole grains, fruit, vegetables, and plant-based protein were common to all indices. Similarly, in a meta-analysis of a posteriori-derived dietary patterns identified in prospective cohort studies, prudent diets associated with reduced risk of coronary heart disease were characterised by increased consumption of fish and poultry, low-fat dairy, fruit, nuts, seeds, and whole grains [19]. The consistency of these findings suggest that there are key foods which constitute a diet to help prevent cardiovascular disease, although the precise structure of healthy diets may vary based on cuisines and dietary preferences.

Similar evidence has been observed for dietary patterns to improve risk factors or biomarkers of cardiovascular disease. In a meta-analysis of randomised controlled trials, Ndanuko et al. [22•] showed that dietary patterns associated with lower blood pressure were characterised by increased consumption of vegetables, fruit, whole grains, legumes, nuts and seeds, and fish and dairy and reduced intake of meat, sweets, and alcohol. Consistent results were identified when a posteriori dietary patterns were explored in recent clinical trials, where dietary patterns characterised by nuts, seeds, fruit, and fish were associated with lower blood pressure in a sample of overweight and obese adults [23, 24]. Further, two meta-analyses found dietary patterns characterised by plant-based foods such as fruits, vegetables, and whole grains to be inversely associated with levels of C-reactive protein, a marker of chronic inflammation [25, 26].

Other areas of science have exposed the association between specific nutrient intakes and cardiovascular disease which may explain the beneficial effects of the foods which characterise the healthy dietary patterns. For example, fruits, vegetables, and whole grains are rich in fibre which may reduce the risk of a number of chronic diseases, including cardiovascular disease [27]. Fruits and vegetables in particular are also rich in phytochemicals such flavanols and anthocyanins which largely contribute to the colours of the flesh and skin [28•]. These compounds have also been associated with reduced cardiovascular disease as well as improvements in mental health, gut health, and cancer risk [29, 30]. Habitual consumption of foods containing polyunsaturated fatty acids such as fish and nuts has also been associated with reduced risk of cardiovascular disease [31, 32]. In addition to the favourable effects of these individual foods, the reduced risk of disease found with these dietary patterns is likely to be due to the synergistic benefits from consuming a whole diet, whereby individual nutrients and foods consumed in combination yield greater benefit than when considering the components in isolation [3••]. Furthermore, the absence or very low intake of foods which may have deleterious effects on health, such as those high in sodium, added sugar, and saturated fat, may also in part explain the benefits observed with ‘healthy’ dietary patterns.

### Establishing Food Groups as Units of Analysis

Dietary pattern analyses whether they follow an a priori or *posteriori* approach are dependent on the ways foods are described and categorised. While more common to a priori methods, grouping of food items is a process that requires a substantial understanding of food composition, food preparation practice/cuisines, and patterns of consumption. At the basic level across many countries, foods are grouped into ‘core’ food groups based on conceptual and compositional similarities. These similarities, however, are underpinned by assumptions and led by a number of rules that are not always articulated, and problems can occur if the research question is not properly aligned. For example, for studies in diet and cardiovascular disease, foods can be categorised together based on their macronutrient composition, given the interest in dietary fat. Statistical methods such as cluster analysis [33, 34] may categorise a cereal food like pasta with a dairy food such as custard given their carbohydrate profiles [35], but this may be a mis-categorisation when the translation of the research is intended to consider foods that could be interchanged within meals. Issues such as consumer understanding of food categories or alignment with dietary guidelines often need to be considered [36]. Other issues include botanical classification food preparation practices or even the colour of the food which may be connected to food components of interest in cardiovascular disease. In some dietary guidelines, for example, sub-grouping of vegetables by colour has been used to encourage variety and considers the compounds contained therein that provide this colour. The red to blue hue of a fruit or vegetable for example is often from the anthocyanin compounds within foods, which need to be included when grouping the foods [29, 30].

While the conceptual understanding of food categorisation or grouping may appear to be straightforward for macronutrient components, problems remain for even broader groupings of foods. For example, legumes are an important source of protein in a vegetarian eating pattern [37] and therefore serve as a meat alternative, but for the omnivorous eating pattern, it may appear as a vegetable [38]. Thus, an alignment of food categorisation with the context of the research question remains important.

The regularity of consumption of different foods in a population is another significant consideration. Dietary pattern analyses of population-wide intakes need to consider the likelihood of varied eating patterns that are inherent to human behaviour [39–41]. Analyses of data on targeted consumer groups based on cultural practices, religious, or ethical beliefs may show less variability by comparison to the broader population, but there can still be heterogeneity. When grouping food items, the purpose of examining the dietary pattern as well as understanding culinary use and consumption patterns remains important [42]. The nutrient composition of the food is usually a central consideration but food preparation practices can influence this. For example, boiling vegetables, for extended periods of time, can lead to substantial losses of vitamin C. Thus, additional data on food preparation may need to be collected when using a food composition database after considering the inherent nested hierarchical food groups that are based on conceptual and compositional data.

### Ongoing Development of Food Composition Databases

Research on food composition analysis underpins much of the development and analysis of dietary patterns. Allowing the translation of food intake data to nutrient consumption data [28•], food composition analysis examines foods from a number of different angles, from the proportion of food that is edible, to the range of nutrients within each food and the level of nutrient retention under varied cooking conditions. While each country and region has their own unique food composition data suited to the environmental and food handling conditions, food composition databases form a foundation component of determining dietary patterns in research with small or large samples of the population.

Despite regional differences, common elements of food composition analysis date back to the measurement of energy contributions from the proximate components of foods, including macronutrients. The basic Atwater conversion factors allowed for the assessment of the relative contributions of macronutrients to the total energy (calorie) value of the food [43•]. While the units are represented differently in various places (calories vs kilojoules), the basic premise of equivalence to the amount of energy attributed to that nutrient has remained constant.

In the analyses of dietary patterns, the energy contribution from individual foods, food groups, or whole diets is often compared. It is important however to consider the differences between using absolute versus relative values for dietary energy within a population group [44, 45]. Measures of absolute intakes do not account for the individual needs of each person nor for physiological differences within the sample which may affect requirements, for example between males and females. Despite this, the contribution of a food to a person’s energy intake is often the first question asked. Energy contributions arise from the fat, protein, carbohydrate, and alcohol components of the foods consumed. These major categories of ‘proximates’ also give rise to what could be considered the next level of food composition analysis including sugars and fibre (with the remainder contributed by ash). For many years, the conversion of absolute values for carbohydrate and protein was accepted at 4 cal (or 16 kJ) [46]. More recently, this was refined to 17 kJ for each with the values for fat and alcohol remaining unchanged. This shift also saw a move to the term ‘available carbohydrate’ in place of total carbohydrate which had been previously used [43•]. There have been a number of changes in food composition practice over time but without an awareness of these changes, researchers may continue to use dated methods or similarly compare outcomes of studies without an understanding [44] of the impact of these changes. The shift in data presentation from available to total carbohydrates continued to use an Atwater-style factor for calculation, though it now considers the relative contributions coming from fibres, starches, and sugars in their varying forms. Although these factors remain as an average for each component, energy for dietary patterns that are high in fibre for example may resultantly be underestimated [47]. Differences in metabolisable energy measured using feeding studies in older persons have also demonstrated an over estimation of energy from Atwater factors compared with measured energy from in-direct calorimetry [48]. Similarly, we now also know about the impact of different forms of processing on the energy contributions of a food. Nuts when consumed whole compared with chopped or roasted compared with natural provide differing contributions to the metabolisable energy [49]. This again showed an overestimation of Atwater factors but related to an individual food type [49, 50] rather than a dietary pattern.

With respect to micronutrients, the listings of traditional vitamin compounds within food composition data have remained unchanged. Many micronutrients undergo chemical analyses undertaken in the laboratory. The recommendation is to follow the AOAC guidelines [51] for the type of analyses undertaken. Advances or refinements in some techniques have meant lower limits of detection, allowing for a nutrient to be found in foods not previously expected. The known ‘gaps’ in the nomenclature of vitamins arose more recently and is based on a consensus of the evidence for the area. Choline [52] for example, previously referred to as vitamin B4, did not have an established deficiency state and was therefore redeemed of its vitamin status. Similar undertakings also occurred for biotin which was previously known as vitamin H and later as B7 [53]. While these compounds have an important role in human physiology, they did not align with the assumptions for inclusion in the vitamin category. At this stage, such components are comprised of their own food composition tables due to the complexity of the compounds. Minerals, organic and inorganic elements, and newly identified compounds such as phytochemicals (where the evidence base is too limited to determine a physiological need or deficiency) may be considered in dietary pattern analyses. For these purposes, food composition databases will change with time as new analytical data become available or as new methods of analysis are developed [54, 55].

The application of food databases to dietary intake data requires the user to understand the differences between the data available in the region of the dietary survey. In Australia and the USA, two food composition databases exist: a survey and a reference database. The survey database was developed to serve population intake data analysis and contains only the foods reported by the population at the time of data collection [43•]. This is most commonly the database used to analyse other dietary data for the region carefully aligned with the timing of the data collection. The use of the incorrect database can result in substantial differences in outcomes for a study [56]. Therefore, upskilling and professional development of persons using food composition data is vital to avoid incorrect use of the data.

The food composition data on food labels also deserves a mention. These data may be useful for creating information on the food supply but they are too limited in scope to provide useful information on dietary patterns [57, 58]. When data are collected at a time of transition for a country, the data may also need to be updated to suit the release of more recent nutrient data. This was seen, for example, in one of our clinical trials where a new process needed to be created to shift data from a 2007 to a 2013 database due to a lack of concordance file [59].

### Applications of Food Categories in Public Health Advocacy

Translating the evidence on dietary patterns to public health policy and practices implicates the food categories that have been utilised in research. Thus, any problematic areas for research are also likely to emerge in practice. The 2015–2020 Dietary Guidelines for Americans (DGA) emphasised healthy dietary patterns inclusive of vegetables, fruits, grains, low-fat dairy foods, protein-rich foods, and oils [60]. A number of dietary patterns, including the Mediterranean pattern, were suggested combining these food categories. Nevertheless, researchers considering a replication of the PREDIMED study in the USA found a number of issues that needed to be considered based on food categorisation and usual consumption patterns, including the choice of culinary oil, the required high intake of vegetables, and the use of sauces such as sofritos [61]. Food groups similar to those in the DGA were referred to in the Australian Dietary Guidelines (ADG) [62]. Associated educational materials referred to ‘discretionary foods’ indicating a wide range of foods that were considered outside the categories recommended in the guidelines. To enable some analyses of the Australian Health Survey, the food databases included a further categorisation of food groups as discretionary or non-discretionary. This system provided a reference point for the Health Star Rating program developed by Australian state and territory governments to indicate a product’s overall nutritional quality, indicating a rating between 0 (lowest) and 5 stars (highest) on the label of packaged foods [63]. Given the discussion above, it is not surprising that issues have been raised regarding the specificity of health star ratings with respect to alignment with foods suggested in the ADG [64]. Finally, a new emerging issue when considering applications of dietary patterns research is that of environmental impacts [65]. It may not be enough to consider the quality of food composition data and the specificity of food categories in identifying and creating healthy diets. The resources required to grow the food and deliver it for consumption with minimal environmental damage may well become another food characteristic for consideration.

## Conclusions

In the last 70 years, exposing the relationship between diet and cardiovascular disease has taken a number of twists and turns, with the focus shifting from dietary patterns to specific nutrients and foods and back again. Today, there is an appreciation that nutrients, foods, and dietary patterns are interdependent concepts, and this needs to be considered in the research effort. With the trend toward more food-based research, statistical methods for assessing dietary patterns have emerged using either a preconceived value system in a priori methods or a more open-ended approach with a posteriori methods. Either way, the identification of similar foods and relative dietary values has helped expose diet quality in a number of populations. In each case, however, the methodology has required a consideration of nutrient-food-diet interdependence, particularly in the classification of food groups.

Knowledge of how foods are categorised within a food composition database, how they are named as well as understanding the data origins within the database should be at the forefront for nutrition researchers undertaking dietary pattern analyses. Without this understanding, the patterns within the diet may not be articulated clearly or aligned with the constantly evolving area of food science. Finally, applications of research on dietary patterns will also need to consider the limitations of the methods available to study them and put the research in context to assure a benefit including and beyond the prevention of cardiovascular disease.
